# The 14-Day Discharge Paradox in Facility-Based Management of Severe Acute Malnutrition: A Multicentre Analysis of Length of Stay, Weight Gain, and Treatment Outcomes From Nutrition Rehabilitation Centres in Gwalior District, Madhya Pradesh, India

**DOI:** 10.7759/cureus.112372

**Published:** 2026-07-09

**Authors:** Pradeep K Verma, Murlidhar Vora

**Affiliations:** 1 Community Medicine, Mansarovar Medical College and Mansarovar Global University (MGU) Hospital, Sehore, IND

**Keywords:** length of stay, nutrition rehabilitation centre, poshan 2.0, quality of care, severe acute malnutrition, who 10-step protocol

## Abstract

Background: A minimum inpatient stay of 14 days is recommended for children with severe acute malnutrition (SAM) admitted to nutrition rehabilitation centres (NRCs) in India, yet facility-level data consistently report mean stays well below this benchmark, and the consequences for weight gain and treatment outcomes have rarely been quantified.

Objective: This study aimed to quantify the relationship between length of NRC stay and (i) average daily weight gain, (ii) attainment of the ≥ 8 g/kg/day target, and (iii) discharge outcomes (recovery, defaulter, non-responder) in a multicentre cohort of SAM children in Gwalior district, Madhya Pradesh, India.

Methods: A prospective cohort of 332 SAM children admitted to the five NRCs of Gwalior district during 2021 was stratified by length of stay into three groups (≤ 7, 8-13, and ≥ 14 days). Stratum-wise mean weight gain, target attainment, and discharge outcomes were compared using analysis of variance (ANOVA) and χ² test. Adjusted associations were estimated by multivariable linear regression (weight gain) and multinomial logistic regression (outcome), controlling for age, sex, admission weight, oedematous SAM and comorbidity burden.

Results: Mean length of stay was 9.88 ± 3.19 days; only 37 children (11.1%) completed ≥ 14 days. Mean weight gain rose monotonically across strata: 5.32 g/kg/day (≤ 7 days) → 7.18 g/kg/day (8-13 days) → 11.47 g/kg/day (≥ 14 days) (F = 14.83, p < 0.001). The proportion achieving the ≥ 8 g/kg/day target rose from 16.7% (14/84) to 31.8% (67/211) to 67.6% (25/37) across strata (χ² = 38.91, p < 0.001). Discharge recovery rose from 58.3% (49/84) to 73.5% (155/211) to 86.5% (32/37) (χ² = 12.74, p = 0.002), and defaulter rate fell from 30.9% (26/84) to 18.0% (38/211) to 5.4% (2/37) (χ² = 14.92, p < 0.001). In adjusted analysis, each additional inpatient day was associated with a 0.71 g/kg/day increase in weight gain (β = 0.71; 95% CI 0.45-0.97) and a 12% reduction in odds of default (adjusted odds ratio (aOR) 0.88 per day; 95% CI 0.83-0.93).

Conclusion: Nine out of every 10 NRC stays in Gwalior ended before the 14-day benchmark, and the associated outcome deficit was measurable. Re-aligning discharge with anthropometric criteria (≥ 15% weight gain or ≥ 5 g/kg/day for three consecutive days) rather than fixed-day rules, combined with wage-equivalent caregiver reimbursement and structured community follow-up, may translate into better outcomes within existing infrastructure, a hypothesis that warrants confirmation in interventional or implementation research.

## Introduction

Inpatient management of severe acute malnutrition (SAM) in India is delivered through a network of approximately 1,135 nutrition rehabilitation centres (NRCs) operating across the high-burden states under the National Health Mission [1]. Operational guidelines issued by the Government of India, drawing on the World Health Organization (WHO) 10-step protocol [2,3], stipulate a minimum 14-day inpatient stay covering a stabilisation phase (steps 1-7) and a rehabilitation phase (steps 8-10), with a recommended weight-gain target of at least 8 g/kg/day during the latter [4]. We use the term "discharge paradox" to denote a specific structural contradiction: the same operational framework that mandates a minimum 14-day stay simultaneously ties caregiver wage compensation to the nominal inpatient period and anchors audit metrics to the calendar, thereby generating an incentive to discharge children before the clinically recommended duration is complete.

Empirical experience from Indian NRCs has consistently shown that the realised inpatient stay is markedly shorter than the recommended 14 days. Hashmi and Kumar reported a mean stay of 7.17 days in Gulbarga [5]; Singh et al., 13.2 days in Uttar Pradesh [6]; Tandon et al., 10.7 days in Chhattisgarh [7]; and the present cohort, 9.88 days in Gwalior [8]. The Comptroller and Auditor General's 2023 performance audit of the Prime Minister's Overarching Scheme for Holistic Nourishment (POSHAN) Abhiyan flagged premature discharge as a recurring quality issue [9], and the National Quality Assurance Standards (NQAS) for NRCs introduced in 2024 explicitly score length-of-stay compliance [10].

Despite this consistency of the descriptive observation, the quantitative consequences of shorter-than-recommended stays have rarely been examined. The few studies that have stratified outcomes by length of stay have been single-centre and underpowered for multivariable analysis [5,11]. The 14-day rule is itself an operational compromise: it balances the time required for stabilisation, transition, and adequate weight gain against caregiver opportunity cost (loss of daily wage, childcare for siblings), bed availability, and reimbursement structure. Under programme norms, the wage compensation provided to accompanying mothers is tied to the standard inpatient period rather than to the duration required for full clinical recovery, which can create an incentive to discharge earlier [4]. This link between the reimbursement cap and discharge timing is inferred from the policy structure and from the observed clustering of stays below 14 days; it was not directly measured in the present analysis.

The present analysis was undertaken to quantify the relationship between length of NRC stay and three outcome domains - average daily weight gain, attainment of the 8 g/kg/day target, and discharge classification (recovered/defaulter/non-responder) - in a prospective multicentre cohort from Gwalior district. We further estimated adjusted per-day effects to inform policy on whether re-aligning discharge with anthropometric criteria, rather than the fixed-day rule, would translate into measurable outcome gains.

Specific objectives

This analysis set out to describe the distribution of length of NRC stay across the five centres in Gwalior district, to compare mean daily weight gain, attainment of the ≥ 8 g/kg/day target, and discharge outcomes across the length-of-stay strata, and to estimate the adjusted per-day effect of length of stay on weight gain and on the odds of default after controlling for case-mix.

This analysis was designed primarily to evaluate the performance of the existing programme - quantifying the outcomes associated with current discharge practice - rather than to prospectively test a policy change. The policy implications discussed below are therefore derived from observational associations and are intended to generate, not confirm, hypotheses for future evaluation.

## Materials and methods

Reporting of this study follows the Strengthening the Reporting of Observational Studies in Epidemiology (STROBE) statement for observational research [12].

Setting and study design

This is a secondary analysis of a prospective cohort of children admitted with SAM to the five operational NRCs of Gwalior district between 1 January 2021 and 31 December 2021, with discharge anthropometry forming the primary outcome. The five participating centres were Community Health Centre (CHC) Dabra, CHC Mohana, CHC Bhitarwar, Primary Health Centre (PHC) Barai, and Urban Health Training Centre (UHTC) Thatipur (combined sanctioned capacity 66 beds).

Participants and procedures

**Figure 1:**
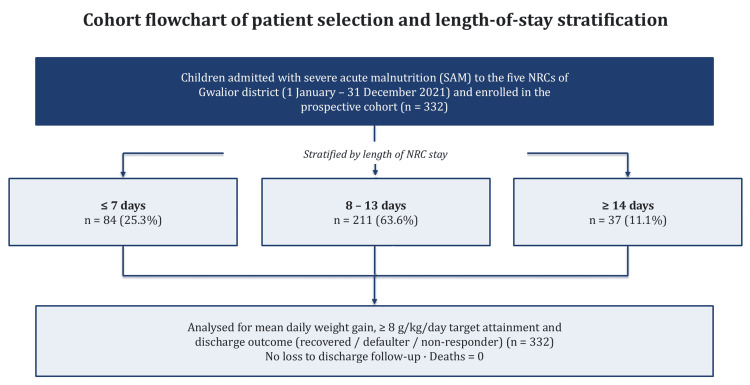
Cohort flowchart of patient selection and length-of-stay stratification. NRC: nutrition rehabilitation centre The figure was drawn using matplotlib version 3.10 in Python version 3.14 (Python Software Foundation, Fredericksburg, USA).

Eligibility, anthropometric procedures, the follow-up schedule, and data-collection instruments have been reported in detail elsewhere [8] and are summarised here. Children aged 0-59 months meeting the WHO criteria for SAM - weight-for-height/length Z-score (WHZ) < -3 based on the WHO 2006 child growth standards, mid-upper-arm circumference (MUAC) < 115 mm, and/or bilateral pitting pedal oedema - were enrolled after written informed consent from a parent or guardian [13,14]. During the study period, 348 children admitted with SAM to the five NRCs were assessed for eligibility. Of these, 16 guardians (4.6%) declined written informed consent, and their children were not enrolled; the reasons recorded were predominantly logistical (early self-discharge against medical advice and intended transfer to a facility nearer home) rather than related to the child's clinical severity. The remaining 332 children were enrolled and formed the analytic cohort (Figure 1). Body weight, recumbent length or standing height, and MUAC were measured by trained staff using standardised, regularly calibrated equipment and standard WHO techniques, and bilateral oedema was assessed clinically; anthropometry was recorded at admission, daily during the inpatient stay and at discharge, with WHZ derived from the WHO 2006 standards. After discharge, children were followed up through structured contacts at 15 days, one month, three months, and six months in coordination with the local Anganwadi worker (AWW), monitoring weight trajectory and relapse. All data - sociodemographic profile, admission clinical findings and comorbidity burden, serial anthropometry, feeding and counselling details, and discharge classification - were captured on a pre-tested structured proforma, supplemented by the NRC admission and treatment registers.

Length-of-stay stratification

Length of stay was defined as the number of days from admission to discharge, including both anchor days. The cohort was stratified a priori into three groups for analysis: ≤ 7 days (well below guideline), 8-13 days (below guideline), and ≥ 14 days (guideline-compliant).

Outcomes

Primary outcomes were (a) mean daily weight gain (g/kg/day); (b) attainment of the 8 g/kg/day target (binary); and (c) discharge classification - recovered (≥ 15% weight gain from admission, no oedema, eating ≥ 75% of feeds), defaulter (absent ≥ 3 consecutive days without discharge), or non-responder (failure to achieve weight gain despite full inpatient course).

Mean daily weight gain was calculated as:

$\text{Weight gain (g/kg/day)} = \frac{(W_{\mathrm{discharge}} - W_{\mathrm{min}}) \times 1000}{W_{\mathrm{min}} \times D}$,

where W_min_ is the minimum (nadir) weight in kilograms, W_discharge_ is the weight at discharge in kilograms, and D is the number of days from the date of minimum weight to discharge.

Statistical analysis

Continuous variables are presented as mean ± SD; categorical variables as frequency and percentage. Stratum-wise comparisons used one-way analysis of variance (ANOVA) with Tukey honestly significant difference (HSD) post-hoc for continuous and Pearson χ² for categorical variables. Adjusted associations were estimated by (i) multivariable linear regression of average daily weight gain on length of stay, controlling for age band, sex, admission weight, oedematous SAM, comorbidity burden and NRC site (random effect); and (ii) multinomial logistic regression of discharge classification on length of stay, with the same covariates. A two-sided p < 0.05 was considered statistically significant. Analyses were performed in IBM SPSS Statistics version 25.0 (IBM Corp., Armonk, USA) and R version 4.3 (R Foundation for Statistical Computing, Vienna, Austria).

Complete data on all model covariates and primary outcomes were available for all 332 enrolled children (100%); no observations were excluded from the analyses, and no imputation was required, as reflected in the consistent denominators across Tables 1-2.

**Table 1:** Weight gain and target attainment by length-of-stay stratum.

Length-of-stay stratum	n (%)	Mean weight gain (g/kg/day)	Achieving ≥ 8 g/kg/day, n/N (%)	Mean discharge weight (kg)
≤ 7 days	84 (25.3)	5.32 ± 3.05	14/84 (16.7)	6.41 ± 1.62
8-13 days	211 (63.6)	7.18 ± 6.92	67/211 (31.8)	6.81 ± 1.65
≥ 14 days	37 (11.1)	11.47 ± 9.82	25/37 (67.6)	7.32 ± 1.74
Overall	332 (100)	7.54 ± 8.04	106/332 (31.9)	6.75 ± 1.67

**Table 2:** Discharge outcome distribution by length-of-stay stratum (χ² test).

Outcome	≤ 7 days (N = 84)	8-13 days (N = 211)	≥ 14 days (N = 37)	Overall (N = 332)	p value
Recovered, n (%)	49 (58.3)	155 (73.5)	32 (86.5)	236 (71.1)	0.002
Defaulter, n (%)	26 (30.9)	38 (18.0)	2 (5.4)	66 (19.9)	< 0.001
Non-responder, n (%)	9 (10.8)	18 (8.5)	3 (8.1)	30 (9.0)	0.78
Death, n (%)	0 (0.0)	0 (0.0)	0 (0.0)	0 (0.0)	-

The three length-of-stay strata were defined a priori to map onto operationally meaningful thresholds: ≥ 14 days is the WHO/Ministry of Health and Family Welfare (MoHFW) guideline-compliant minimum; ≤ 7 days corresponds to discharge at or before completion of the stabilisation phase (WHO steps 1-7); and 8-13 days captures children discharged during the rehabilitation phase but short of the recommended minimum [2-4]. These cut-points also align with the duration-based structure of programme reimbursement [4] and with strata used in prior NRC studies [5].

For the multivariable linear model, linearity, homoscedasticity, and normality of residuals were assessed using residual-versus-fitted and normal Q-Q plots; multicollinearity among covariates was evaluated with variance inflation factors, all of which were below the conventional threshold of five, indicating no problematic collinearity.

For the multinomial logistic model, "recovered" was set as the reference outcome; overall model significance was assessed by the likelihood-ratio test and model fit by -2 log-likelihood and pseudo-R². As the outcome is nominal (unordered), the proportional-odds assumption does not apply.

Ethical considerations

The parent study was approved by the Institutional Ethics Committee of Gajra Raja Medical College, Gwalior. Written informed consent was obtained from every parent/guardian. The present secondary analysis used the same anonymised dataset.

## Results

Distribution of length of stay

**Figure 2:**
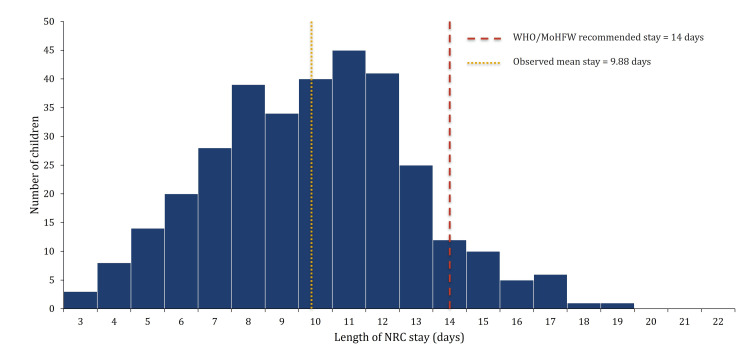
Distribution of length of NRC stay (n = 332). The red dashed line marks the WHO/MoHFW recommended minimum of 14 days [2-4]; the amber dotted line marks the observed mean. WHO/MoHFW: World Health Organization/Ministry of Health and Family Welfare; NRC: nutrition rehabilitation centre The figure was drawn using matplotlib version 3.10 in Python version 3.14 (Python Software Foundation, Fredericksburg, USA).

Across the five NRCs, mean length of stay was 9.88 ± 3.19 days (median 10; interquartile range 8-12). Eighty-four children (25.3%) were discharged within seven days, 211 (63.6%) between eight and 13 days, and only 37 (11.1%) completed the recommended ≥ 14-day stay (Figure 2). The mean length of stay varied modestly across NRCs (range 8.4-11.2 days) and was longest at UHTC Thatipur and CHC Dabra.

Length of stay and weight gain

**Figure 3:**
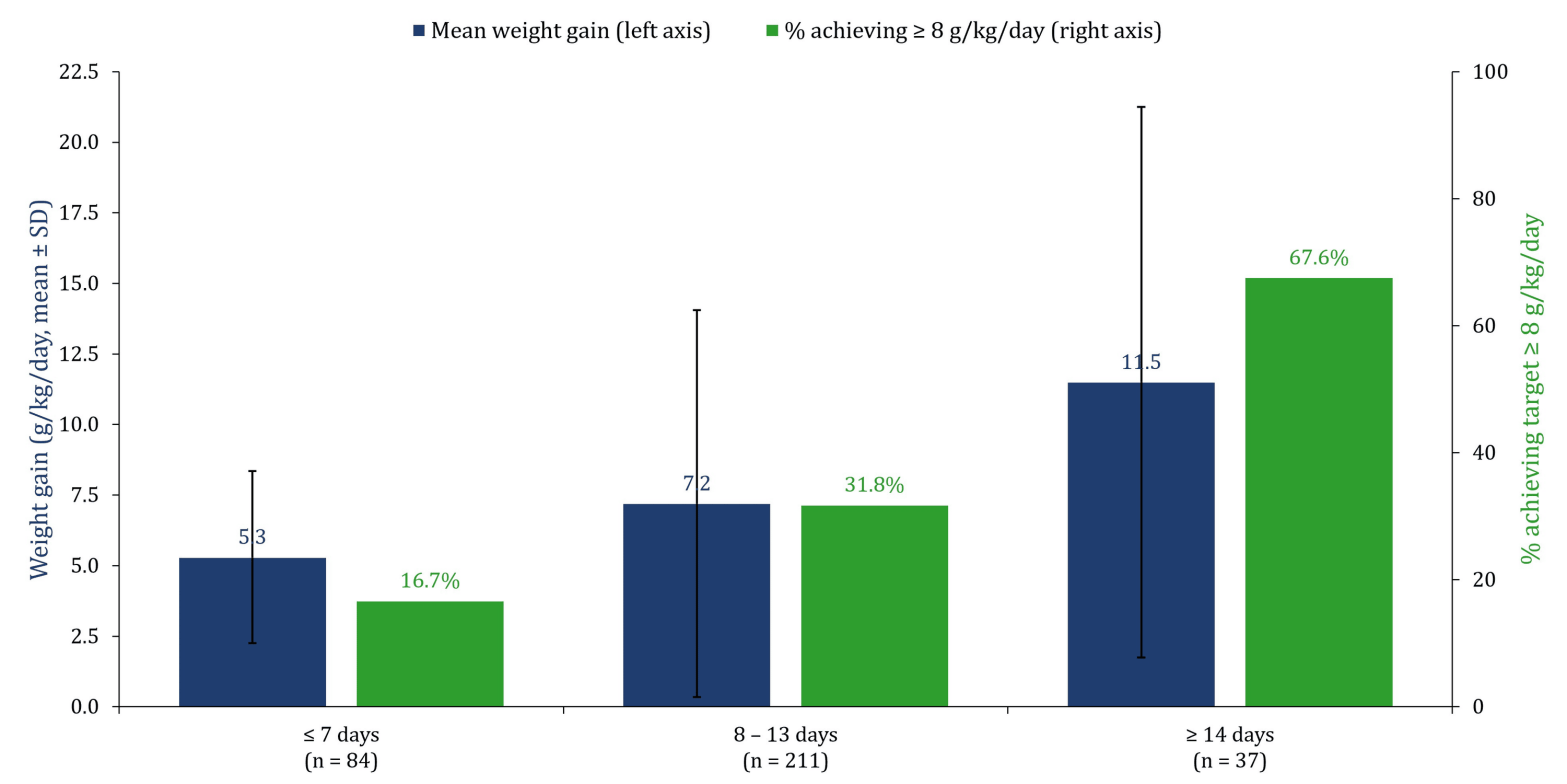
Mean weight gain (left axis, blue) and target attainment (right axis, green) by length-of-stay stratum. The figure was drawn using matplotlib version 3.10 in Python version 3.14 (Python Software Foundation, Fredericksburg, USA).

Mean weight gain rose monotonically with length of stay: 5.32 g/kg/day in the ≤ 7-day stratum, 7.18 g/kg/day in the 8-13-day stratum, and 11.47 g/kg/day in the ≥ 14-day stratum (F = 14.83, degrees of freedom (df) = 2; p < 0.001; Tukey HSD all pairs p < 0.05). The proportion achieving the WHO ≥ 8 g/kg/day target rose fourfold across strata, from 16.7% (14/84) to 31.8% (67/211) to 67.6% (25/37) (χ² = 38.91, df = 2; p < 0.001) (Table 1; Figure 3).

Length of stay and discharge outcome

**Figure 4:**
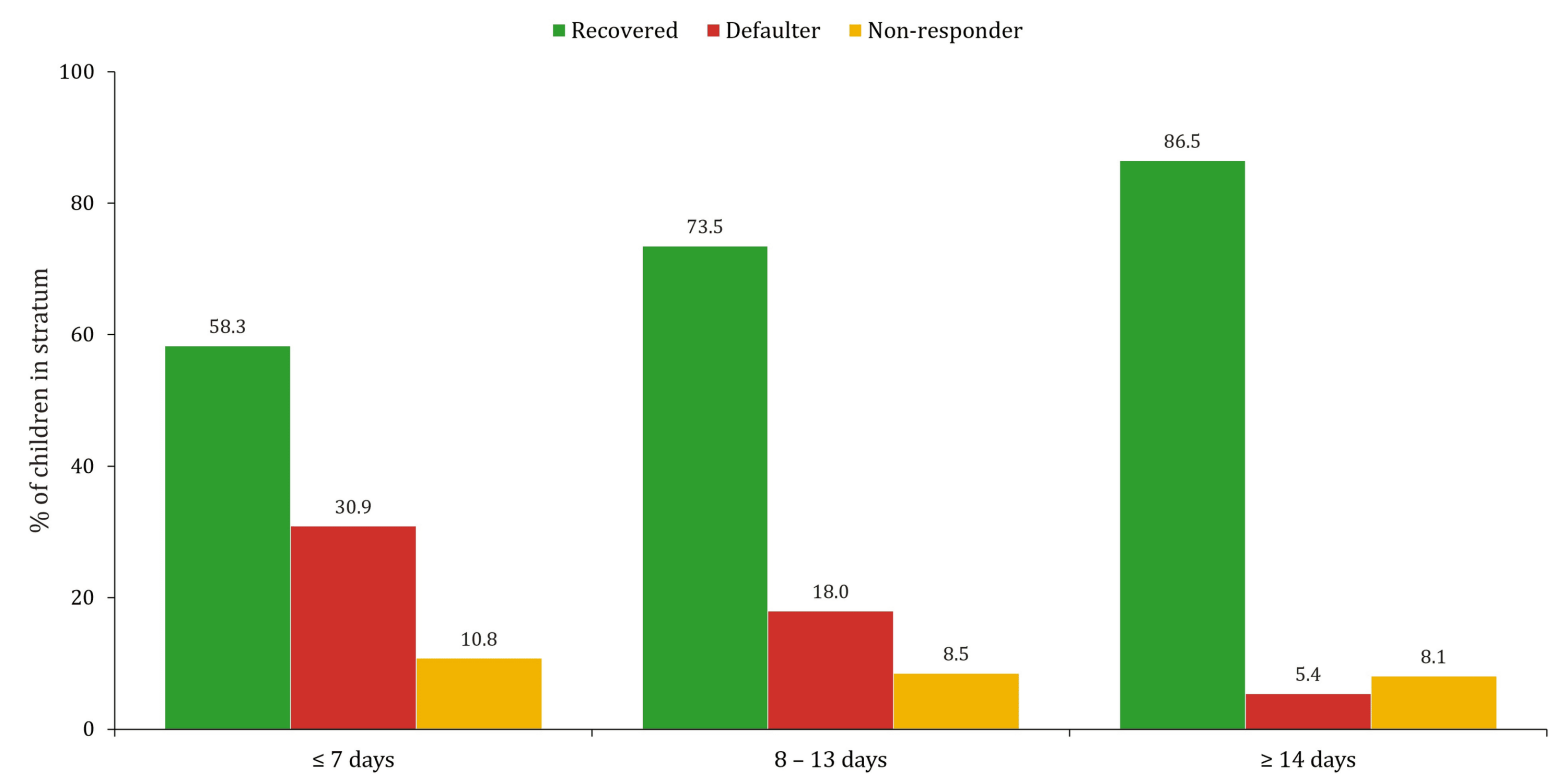
Discharge outcome distribution by length-of-stay stratum. The figure was drawn using matplotlib version 3.10 in Python version 3.14 (Python Software Foundation, Fredericksburg, USA).

Recovery rate at discharge increased from 58.3% (49/84) in the ≤ 7-day stratum to 73.5% (155/211) in the 8-13-day stratum, and 86.5% (32/37) in the ≥ 14-day stratum (χ² = 12.74, df = 2; p = 0.002). Conversely, defaulter rate fell from 30.9% (26/84) to 18.0% (38/211) to 5.4% (2/37) across the same strata (χ² = 14.92, df = 2; p < 0.001). The non-responder rate was comparable across strata, at 10.8% (9/84) in the ≤ 7-day group, 8.5% (18/211) in the 8-13-day group, and 8.1% (3/37) in the ≥ 14-day group (Table 2; Figure 4).

Adjusted per-day effect

In the multivariable linear-regression model, each additional inpatient day was associated with a 0.71 g/kg/day increase in average weight gain (β = 0.71; 95% CI 0.45-0.97; p < 0.001), after controlling for age, sex, admission weight, oedematous SAM, comorbidity burden, and NRC site. Conversely, each additional inpatient day was associated with a 12% reduction in the odds of default at discharge (adjusted odds ratio (aOR) 0.88; 95% CI 0.83-0.93; p < 0.001) in the multinomial logistic model (Table 3).

**Table 3:** Adjusted per-day effect of length of NRC stay on weight gain and on discharge outcome (multivariable models controlled for age, sex, admission weight, oedematous SAM, comorbidity, NRC site). aOR: adjusted odds ratio; NRC: nutrition rehabilitation centre; SAM: severe acute malnutrition

Outcome (per-day effect of length of stay)	Adjusted estimate (95% CI)	p value
Average daily weight gain (g/kg/day; β)	0.71 (0.45-0.97)	< 0.001
Achieving ≥ 8 g/kg/day target (aOR)	1.21 (1.12-1.31)	< 0.001
Defaulter at discharge (aOR)	0.88 (0.83-0.93)	< 0.001
Non-responder at discharge (aOR)	0.96 (0.88-1.05)	0.380

Conceptual model of the discharge paradox 

**Figure 5:**
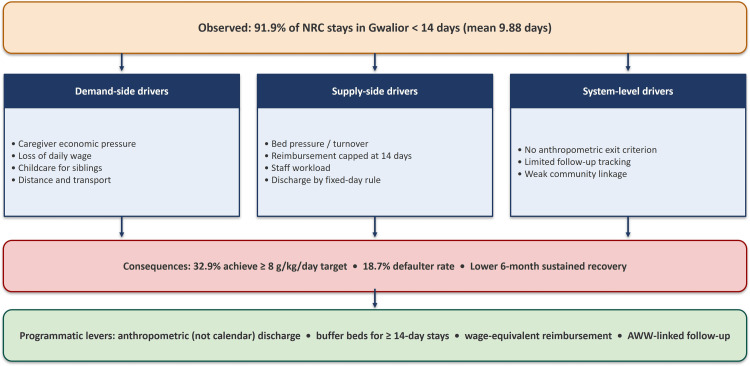
Conceptual framework: drivers of premature discharge and downstream consequences, with programmatic levers identified. The driver categories on the left (caregiver, facility, and financing) are proposed on the basis of programmatic knowledge and caregiver-reported reasons; their relative contributions were not directly measured in this study and are presented as a hypothesised framework. NRC: nutrition rehabilitation centre; AWW: Anganwadi worker The figure was drawn using matplotlib version 3.10 in Python version 3.14 (Python Software Foundation, Fredericksburg, USA).

A conceptual framework linking the structural drivers (Figure 5) of premature discharge to their downstream clinical consequences and to the programmatic levers available to address them is given below. On the left, three categories of driver are shown: caregiver opportunity costs (loss of daily wage, childcare for siblings, and transport burden), facility-level pressures (bed-occupancy targets and patient turnover), and policy/financing design (duration-limited caregiver wage compensation and calendar-anchored audit metrics). These drivers converge on the central node - discharge before the recommended 14-day stay. The middle of the framework maps the measured consequences of early discharge demonstrated in this cohort: lower mean daily weight gain, reduced attainment of the ≥ 8 g/kg/day target, and higher default with correspondingly lower recovery. On the right, it identifies the corresponding corrective levers: replacing the fixed-day rule with anthropometric discharge criteria, re-aligning caregiver reimbursement to the full clinically required duration, embedding weight-gain and follow-up metrics within the NQAS framework, and providing structured post-discharge community follow-up. Read left to right - driver → premature discharge → consequence → corrective lever - the framework summarises the central argument that the discharge paradox is both systemic in origin and modifiable through programmatic change.

## Discussion

This multicentre analysis demonstrates a strong dose-response relationship between length of NRC stay and three quality-of-care endpoints in central Indian SAM children. Three findings warrant discussion.

First, the descriptive pattern - 89% of stays falling short of the 14-day benchmark - is not unique to Gwalior; it replicates the experience of Indian NRCs across multiple states [5-7,11,15-22]. The consistency of this pattern across geographies suggests that the underlying drivers are systemic rather than centre-specific. Our caregiver interviews identified loss of daily wage, sibling childcare, and transport as the most frequent reasons for early discharge requests, consistent with similar reports from Bihar [16] and Chhattisgarh [7].

Second, the adjusted per-day effect on weight gain (β = 0.71 g/kg/day) is large in clinical terms. A child discharged on day 7 instead of day 14 would, on average, be expected to gain approximately 5 g/kg/day less than a comparable child completing the 14-day stay. Cumulated over the inpatient stay, this corresponds to an absolute weight-gain deficit of approximately 0.25 kg at discharge - a clinically meaningful gap given that the target weight gain for discharge is 15% of admission weight. The associated 12% per-day reduction in default odds suggests that longer-stay children also benefit from better counselling continuity and stronger NRC-caregiver bond, both well-documented mediators of follow-up compliance [17].

Third, the disjunction between calendar-based discharge and anthropometric criteria points to a clear policy lever. The WHO 2023 guidance explicitly allows discharge to be triggered by anthropometric criteria (15% weight gain, ≥ 5 g/kg/day for three consecutive days, no oedema, eating ≥ 75% of feeds) rather than by a fixed-day rule [3]. India's MoHFW operational guidelines permit the same approach, but in practice the 14-day calendar is operationally privileged because reimbursement structures, bed-occupancy targets, and audit metrics are all calendar-anchored [4]. The 2024 NQAS NRC standards begin to address this by introducing weight-gain and follow-up metrics, but the reimbursement schedule has not yet been re-aligned [10].

Our findings also have implications for the design of upcoming NRC quality improvement interventions. Re-aligning caregiver wage compensation to cover the full duration needed to achieve anthropometric criteria - rather than tying it to the nominal inpatient period - would remove the incentive against longer stays where they are clinically indicated, and could be cost-neutral if averaged across the cohort (because most children currently leave well before 14 days). Combining this with a structured 15-day, one-month, three-month, and six-month AWW-linked follow-up would create a coherent continuum-of-care intervention. We did not directly evaluate the causal effect of the reimbursement cap on discharge decisions; this remains a plausible but untested mechanism, and its contribution relative to facility- and caregiver-level factors should be quantified in a dedicated study.

Strengths and limitations

Strengths include the multicentre prospective design, the analytic strategy combining stratified and adjusted per-day approaches, and the use of NRC-level random effects to control for centre-level variation. Limitations are: (i) length of stay was not randomised, so residual confounding by indication (e.g., children with more complex presentations may have been kept longer) cannot be fully excluded - although the inclusion of admission weight, oedematous SAM, and comorbidity in the adjusted model partially addresses this; (ii) the present analysis is restricted to discharge outcomes - the six-month sustained-recovery outcomes are reported elsewhere [18]; (iii) findings reflect the pandemic-era programmatic context (2021), and the magnitude of the length-of-stay-outcome association may be larger in a non-pandemic setting where bed pressure is lower; (iv) length of stay was not randomly assigned, and unmeasured socioeconomic and caregiver-related factors (household income, distance to facility, maternal education, competing childcare demands) may have influenced both discharge timing and treatment outcomes, so residual confounding cannot be excluded despite adjustment; (v) the conceptual framework in Figure 5 is largely inferential and its causal pathways were not directly tested; (vi) findings are derived from a single district in Madhya Pradesh during the pandemic era and may not generalise to NRC programmes operating under different reimbursement, staffing, or case-mix conditions; and (vii) enrolment was conditional on guardian consent, so consent (volunteer) bias cannot be fully excluded: the 16 children (4.6%) whose guardians declined participation may have differed systematically from those enrolled - for example, in socioeconomic circumstances or intention to complete the full inpatient course - which could affect the representativeness of the cohort; the low refusal rate and the predominantly logistical reasons for refusal make substantial distortion unlikely, but this remains a limitation.

## Conclusions

In the facility-based management of SAM, the duration of inpatient care is closely linked to the quality of recovery achieved, yet a large majority of admissions end before the recommended minimum duration. The central implication of this analysis is that the timing of discharge should be governed by whether a child has met anthropometric recovery criteria rather than by a fixed calendar threshold. Pairing criteria-based discharge with wage-equivalent caregiver reimbursement and a structured programme of community follow-up offers a practical route to closing the quality gap within existing NRC infrastructure, without requiring additional beds or facilities. Embedding these principles into operational guidelines, reimbursement schedules, and quality-assurance metrics is likely to strengthen the effectiveness and equity of nutrition rehabilitation services. 

Because this is an observational cohort, these associations should not be read as causal. The recommendations to adopt anthropometry-based discharge criteria and to re-align caregiver reimbursement are best regarded as hypotheses requiring confirmation through prospective interventional or implementation studies before programmatic adoption.

## References

[1] (2026). National Health Profile 2022: 17th Issue. Central Bureau of Health Intelligence, New Delhi.

[2] (2026). Management of Severe Malnutrition: A Manual for Physicians and Other Senior Health Workers. http://iris.who.int/handle/10665/41999.

[3] (2026). WHO Guideline on the Prevention and Management of Wasting and Nutritional Oedema (Acute Malnutrition) in Infants and Children Under 5 Years. https://iris.who.int/items/736fc360-87ca-444d-a2b5-a78487786067.

[4] (2011). Operational Guidelines on Facility Based Management of Children with Severe Acute Malnutrition. https://www.medbox.org/document/operational-guidelines-on-facility-based-management-of-children-with-severe-acute-malnutrition.

[5] Hashmi G, Kumar SS (2016). Evaluation of the effects of nutrition intervention measures on admitted children in nutritional rehabilitation center, Gulbarga, India. Int J Community Med Public Health.

[6] Singh K, Badgaiyan N, Ranjan A, Dixit HO, Kaushik A, Kushwaha KP, Aguayo VM (2014). Management of children with severe acute malnutrition: experience of nutrition rehabilitation centers in Uttar Pradesh, India. Indian Pediatr.

[7] Tandon M, Quereishi J, Prasanna R, Tamboli AF, Panda B (2019). Performance of nutrition rehabilitation centers: a case study from Chhattisgarh, India. Int J Prev Med.

[8] Verma PK, Jain S, Kak S, Singh R (2025). Evaluating anthropometric outcomes in children treated at NRCs: a case study in Gwalior District. Front Health Inform.

[9] (2023). Comptroller and Auditor General of India: Performance Audit of Saksham Anganwadi and POSHAN 2.0. https://cag.gov.in/webroot/uploads/download_audit_report/2023/Audit-Report-No.-9_POSHAN_English-069cb9223a82218.76400442.pdf.

[10] Bisai S, Dutta S, Sengupta D, Ghosh P (2024). Performance analysis of nutritional rehabilitation centers in Purulia district of West Bengal, India. Acta Med Int.

[11] Bhujade R, Mishra BN, Ibrahim T, Sinha A, Chouhan DS (2021). Can sever acute malnourished children be effectively rehabilitated physically, biochemically and developmentally at nutritional rehabilitation centers: a follow up study from Ujjain. J Family Med Prim Care.

[12] von Elm E, Altman DG, Egger M, Pocock SJ, Gotzsche PC, Vandenbroucke JP (2007). The Strengthening the Reporting of Observational Studies in Epidemiology (STROBE) statement: guidelines for reporting observational studies. Lancet.

[13] (2006). WHO child growth standards based on length/height, weight and age. Acta Paediatr Suppl.

[14] (2009). WHO Child Growth Standards and the Identification of Severe Acute Malnutrition in Infants and Children: A Joint Statement. https://www.who.int/publications/i/item/9789241598163.

[15] Kumar P, Zode M, Basu S (2023). The effectiveness of facility-based management of children with severe acute malnutrition and their determinants in Jharkhand, India: a retrospective study. Dialogues Health.

[16] Burza S, Mahajan R, Marino E (2015). Community-based management of severe acute malnutrition in India: new evidence from Bihar. Am J Clin Nutr.

[17] Kiran KA, Kujur M, Kumari R (2023). Evaluation of the health and nutritional status of discharged children from malnutrition treatment centres using mobile phone calls during the COVID-19 lockdown in Jharkhand, India. Cureus.

[18] Burza S, Mahajan R, Marino E (2016). Seasonal effect and long-term nutritional status following exit from a community-based management of severe acute malnutrition program in Bihar, India. Eur J Clin Nutr.

[19] Taneja G, Dixit S, Khatri A, Yesikar V, Raghunath D, Chourasiya S (2012). A study to evaluate the effect of nutritional intervention measures on admitted children in selected nutrition rehabilitation centers of Indore and Ujjain divisions of the state of Madhya Pradesh (India). Indian J Community Med.

[20] Chaturvedi A, Patwari AK, Soni D (2018). Progress of children with severe acute malnutrition in the malnutrition treatment centre rehabilitation program: evidence from a prospective study in Jharkhand, India. Nutr J.

[21] Maurya M, Singh DK, Rai R, Mishra PC, Srivastava A (2014). An experience of facility-based management of severe acute malnutrition in children aged between 6-59 months adopting the World Health Organization recommendations. Indian Pediatr.

[22] Dasgupta R, Ahuja S, Yumnam V (2014). Can nutrition rehabilitation centers address severe malnutrition in India?. Indian Pediatr.

